# Machine Learning Identifies High-Risk Suicide Profiles in a Population-Based Forensic Registry

**DOI:** 10.3390/diagnostics16162615

**Published:** 2026-08-18

**Authors:** Alin Ionut Piraianu, Anisia-Luiza Culea-Florescu, Elena Stamate, Ana Fulga, Doriana Iancu, Octavian Stefan Patrascanu, Iuliu Fulga

**Affiliations:** 1 Research Centre in the Medical-Pharmaceutical Field, Faculty of Medicine and Pharmacy, “Dunarea de Jos” University of Galați, 800216 Galați, Romania; alin.piraianu@ugal.ro (A.I.P.); ana.fulga@ugal.ro (A.F.); c_doriana@yahoo.com (D.I.); octav974@gmail.com (O.S.P.); iuliu.fulga@ugal.ro (I.F.); 2 Research Center for Electronics, Information Technology and Communications, Faculty of Automation, Computers, Electrical and Electronic Engineering, “Dunarea de Jos” University of Galați, 800201 Galați, Romania

**Keywords:** suicide, forensic medicine, machine learning, random forest, cluster analysis, hanging, medico-legal registry, Romania, suicide prevention

## Abstract

**Background:** Suicide is a leading cause of preventable death, yet machine learning (ML) analyses of forensic (medico-legal) suicide data are scarce and, to our knowledge, absent for Romania. Population-based forensic registries offer exhaustive, autopsy-confirmed coverage that is structurally distinct from clinical or civil death-registration data. We applied supervised and unsupervised ML to a complete regional medico-legal suicide registry to profile the method of death and to identify latent victim subgroups of preventive relevance. **Methods:** We analysed 395 consecutive suicide deaths (Galați and Brăila counties, ≈750,000 inhabitants; 2018–2024). Two supervised classifiers—L2-regularised logistic regression (LR) and random forest (RF, 200 trees)—were trained to discriminate hanging from other methods, using eleven sociodemographic and clinical predictors, and evaluated by 10-fold stratified cross-validation. Given severe class imbalance, the area under the ROC curve (AUC) was the primary metric. Model hyperparameters were fixed a priori, and no class-imbalance correction was applied; both decisions are pre-specified and justified in the Methods. Robustness was assessed by stratified non-parametric bootstrap confidence intervals for the odds ratios, a tipping-point sensitivity analysis for the undocumented clinical fields, and Ward-linkage hierarchical clustering as an independent partitioning check. Predictor importance was quantified by out-of-bag (OOB) permutation importance and Spearman correlations. Unsupervised structure was assessed by K-means clustering (k = 2–7), with the optimal solution selected by the average silhouette coefficient and the elbow (WCSS) criterion. Reporting followed TRIPOD+AI and STROBE. **Results:** The study population was predominantly male (87.1%) and rural (73.2%), with a mean age of 54.1 years; hanging accounted for 94.2% of deaths—far above the European average (≈50%). RF achieved AUC = 0.865 ± 0.181 and LR AUC = 0.847 ± 0.192, both within the “excellent” discrimination band; sensitivity was very high (0.995–0.997) and specificity was limited (0.233–0.367), an expected consequence of imbalance. Prior suicide attempts (OOB importance 0.959; Spearman ρ = −0.549, *p* < 0.001; OR = 0.496, 95% CI 0.25–0.72) and the presence of a suicide note (importance 0.575; ρ = −0.439, *p* < 0.001; OR = 0.549, 95% CI 0.34–0.76) were the dominant predictors. K-means identified two well-separated clusters (silhouette = 0.707), and the partition was reproduced exactly by Ward-linkage hierarchical clustering (adjusted Rand index = 1.000). Cluster 2 (*n* = 19; 4.8%) was a clinically distinct, younger subgroup (42.4 vs. 54.7 years) characterised by prior attempts (57.9% vs. 0%), suicide notes (68.4% vs. 0%), higher psychiatric comorbidity (52.6% vs. 30.9%) and lower hanging proportion (36.8% vs. 97.1%)—an exploratory, hypothesis-generating profile of recurrent suicidal behaviour with documented prior contact with the medical or medico-legal system. The principal findings were stable across all plausible degrees of clinical under-documentation in the tipping-point sensitivity analysis. **Conclusions:** ML applied to a complete forensic suicide registry reproduced known regional epidemiology and, beyond classical statistics, isolated an exploratory but clinically coherent high-risk subgroup of direct relevance to the audit of structured post-attempt follow-up. This is, to our knowledge, the first ML study of Romanian forensic suicide data and supports integrating ML into medico-legal research and into the regional targeting and audit of existing post-attempt follow-up provision.

## 1. Introduction

Suicide remains a major and largely preventable public-health burden. The World Health Organization estimated that more than 700,000 people died by suicide worldwide in 2019, corresponding to roughly one in every hundred deaths, with the WHO European Region above the global average [1]. Within Europe, the former socialist countries of the east and south-east carry a disproportionate share of the burden, and Romania is no exception: national mortality data indicate persistently elevated male and rural suicide rates, with a male-to-female ratio well above the western-European average [1,2,3].

The method of suicide is not merely a descriptive detail; it encodes information about intent, lethality, impulsivity, and access to means, and it is central to means-restriction strategies [4]. In Eastern Europe, hanging is the predominant method, typically accounting for 70–80% of male suicides, already far above the pan-European average of roughly one-half reported in large multi-country analyses [2,3]. Method distribution therefore constitutes a strong, regionally specific epidemiological signal that is rarely examined with modern analytical tools.

Machine learning (ML) has rapidly evolved into one of the most influential analytical approaches in modern medicine, supporting disease prediction, diagnosis, prognostic modelling, precision medicine, and clinical decision-making across a wide range of medical specialties. Recent advances have demonstrated the growing clinical utility of artificial intelligence in both chronic and acute conditions, particularly through predictive modelling and risk stratification, highlighting its increasing integration into routine medical research and practice across multiple disciplines, including cardiovascular medicine [5,6,7]. During the last decade, ML methods have also been increasingly applied to suicide research, most successfully using large national healthcare registries and administrative databases [8,9,10,11,12]. These studies have demonstrated good predictive performance for suicide risk, while unsupervised learning techniques have identified clinically relevant subgroups that are often not detected using conventional statistical approaches [8,9,10,11,12].

Within that body of work, ML has been applied across the full spectrum of suicidal behaviour, suicidal ideation, attempted suicide and completed suicide, using clinical records, administrative data and the content analysis of warning signs [13,14]. The forensic approach developed here is complementary to those efforts rather than competing with them, and the complementarity is worth stating explicitly because it is the principal methodological argument for the design. Prospective models operate forwards: they observe a living cohort and estimate who will subsequently engage in suicidal behaviour, and they are necessarily constrained by what is recorded before the event and by the low base rate of the outcome. A complete forensic registry permits the inverse operation. Because every case in it is a completed suicide established at autopsy, and because ascertainment within the catchment is exhaustive rather than sampled, the registry allows risk characteristics to be back-extrapolated from the terminal event to the ante-mortem configuration that preceded it, with no loss to follow-up and no outcome misclassification. The two directions are epistemically independent and mutually constraining: a risk profile that emerges prospectively from a clinical cohort and retrospectively from an exhaustive death registry is considerably better supported than one derived from either alone. Forensic ML should therefore be read as a validation and targeting instrument for prevention research, not as a substitute for prospective prediction.

However, the overwhelming majority of published ML studies rely on clinical, administrative or survey-based datasets. ML analyses of forensic (medico-legal) suicide data—that is, data derived directly from mandatory autopsy of violent or suspected-violent deaths—remain scarce internationally [15,16], and, to our knowledge, none have been published for Romania. A targeted search of indexed databases (PubMed, Scopus, and Web of Science) combining the terms “machine learning”, “suicide”, “Romania”, and “forensic” returned no relevant original studies.

This gap is consequential. Forensic registries possess properties that clinical and civil-registration data lack: they are population-based and exhaustive within a defined jurisdiction (every death within the catchment is examined, with no loss to follow-up), the cause and manner of death are objectively established at autopsy, and toxicological and pathological findings are recorded uniformly [15,16]. These features make forensic registries an underexploited substrate for data-driven profiling, despite the well-known limitation that ante-mortem clinical variables (e.g., psychiatric history) are incompletely documented for decedents who had limited prior contact with health services.

**Figure 1:**
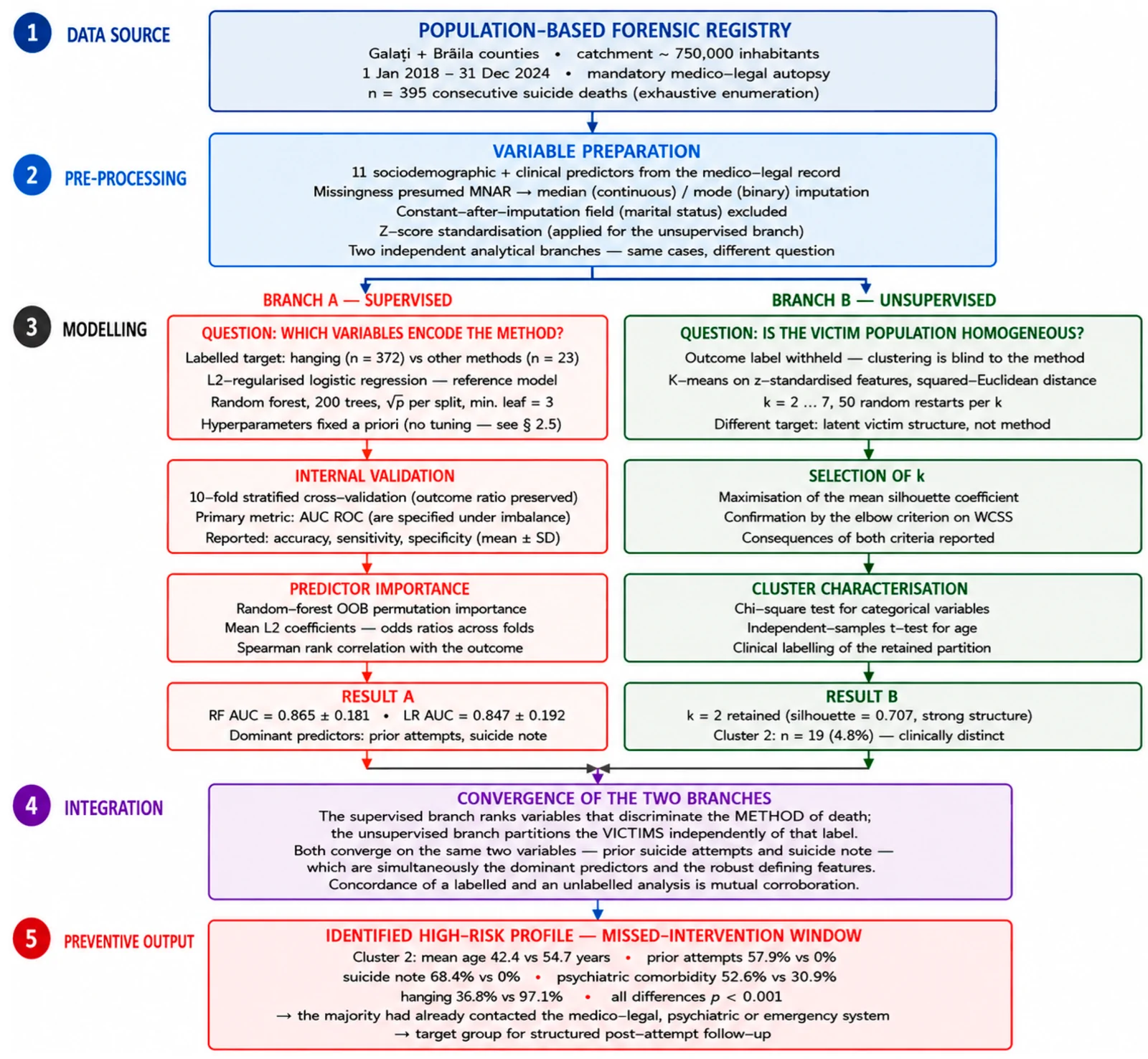
Analytical workflow of the study. Stage 1 defines the population-based forensic source; Stage 2 the pre-processing common to both analytical branches; Stage 3 the two branches, which use the same cases but pose different questions—Branch A (supervised) takes the method of death as a label and asks which variables encode it, Branch B (unsupervised) withholds that label and asks whether the victim population is homogeneous; Stage 4 the integration of the two, and Stage 5 the preventive interpretation.

Against this background, we applied a combined supervised and unsupervised ML framework to a complete regional medico-legal suicide registry covering two south-eastern Romanian counties over seven consecutive years. Our objectives were threefold: (i) to quantify the discriminative information that routinely recorded forensic variables carry about the method of death, using random forest and regularised logistic regression [17,18]; (ii) to rank predictor importance with permutation-based and correlational methods [19]; and (iii) to test, without supervision, whether the victim population is internally homogeneous or organises into distinct subgroups of preventive relevance [20,21]. We hypothesised that, beyond reproducing established regional epidemiology, ML would isolate a clinically meaningful minority profile identifiable before death. The complete analytical pipeline, including the pre-processing common to both stages, the point at which the two branches separate, and the explicit transition from the supervised to the unsupervised analysis, is summarised in Figure 1.

## 2. Materials and Methods

### 2.1. Study Design and Setting

This was a retrospective, population-based registry study. The catchment comprised Galați and Brăila counties in south-eastern Romania (combined population ≈ 750,000). Under the Romanian Code of Criminal Procedure, forensic (medico-legal) autopsy is mandatory in all violent and suspected-violent deaths, including suicide; consequently, the regional medico-legal records constitute an exhaustive, geographically and temporally complete enumeration of suicide deaths rather than a convenience sample. This design confers the methodological status of a population-based registry study, considered superior to sample-based designs for forensic and epidemiological inference.

### 2.2. Data Source and Case Ascertainment

**Figure 2:**
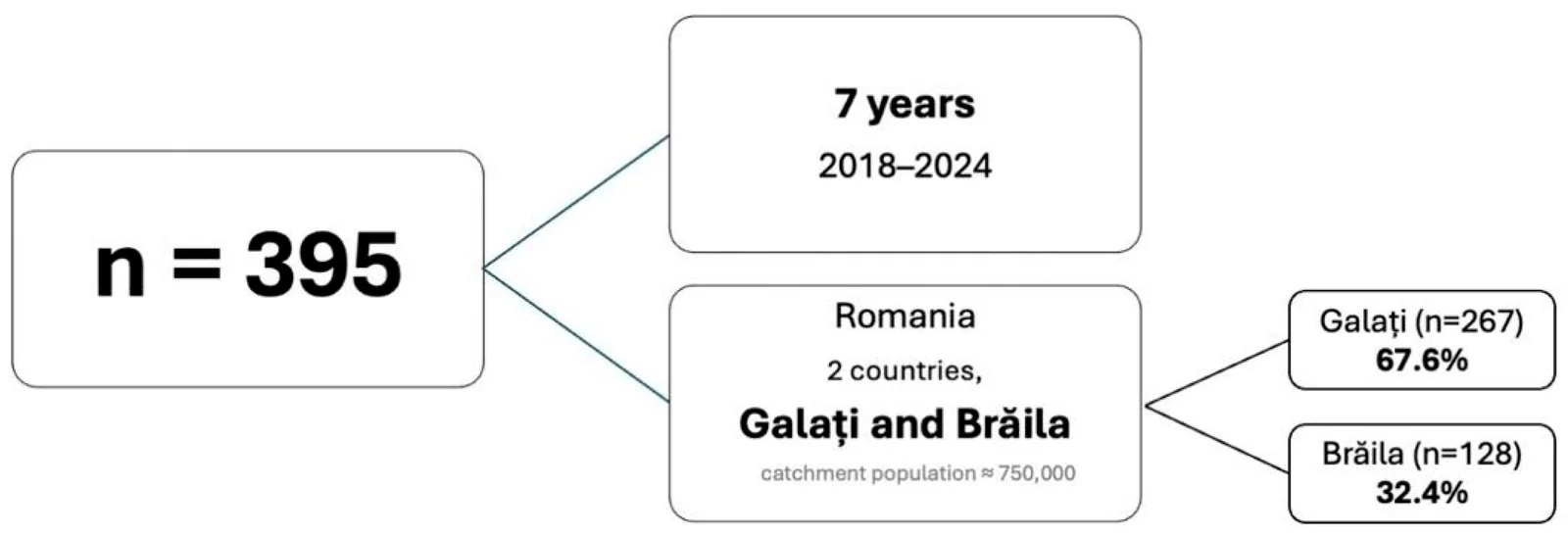
Study population and data source.

All consecutive suicide deaths recorded between 1 January 2018 and 31 December 2024 were extracted, yielding 395 cases (Galați 267, 67.6%; Brăila 128, 32.4%) (Figure 2). The observation window deliberately spans the COVID-19 pandemic, which is exploited in a companion interrupted-time-series study. Records were fully anonymised before analysis.

### 2.3. Who Designates a Case as Forensic: Ascertainment and Potential for Selection Bias

Because every case analysed here entered the dataset by being designated “forensic”, the mechanism of that designation determines what the registry can and cannot represent, and it requires an explicit statement. Under the Romanian Code of Criminal Procedure, the decision is not discretionary and does not rest with the family, the certifying physician or the forensic institute. The medico-legal autopsy is ordered by the prosecutor in every violent, suspected-violent, sudden or otherwise unexplained death, and the institute has no authority to decline a case so ordered. This removes the principal mechanism by which selection bias enters forensic case series elsewhere: case-by-case triage by the examining institution according to interest, workload or family preference, and it is the reason the series can be described as exhaustive rather than sampled.

Two residual mechanisms nevertheless remain, and both are consistent with the reviewer’s concern about the unusually high male-to-female ratio. The first operates upstream of the prosecutor. Ascertainment depends on a death being flagged as suspicious at the scene; a death that raises no external suspicion, most plausibly a fatal intoxication in an elderly or chronically ill decedent, where a plausible natural cause is already on the record, may be certified as natural and never enter the medico-legal system at all. Deaths of this type are disproportionately female and disproportionately non-violent in method. This mechanism would therefore inflate both the male-to-female ratio and the hanging proportion reported here, and it is the more likely explanation for the 6.7:1 ratio than a true regional excess of that magnitude. Accordingly, that ratio should be read as an upper bound rather than as a point estimate, and the same caution applies to the 94.2% hanging share, which is a proportion of ascertained cases rather than of all suicides in the catchment. Whether stigma or the avoidance of family embarrassment contributes to non-referral cannot be determined from within the registry, and we make no claim about it.

The second mechanism concerns uniformity of scene investigation. Investigative resources are not identical between urban and rural jurisdictions, and a death with an ambiguous scene may be resolved with less depth in a rural setting than in a city. The mandatory character of the autopsy order limits this to a question of case detection rather than of case classification: once a case is referred, the examination is conducted to a uniform protocol at the same two regional institutes, so a rural case is not investigated less thoroughly than an urban one at the medico-legal stage. Since 73.2% of the series is rural, any residual detection differential would again act in the direction of under-ascertaining the less obvious methods.

Both mechanisms therefore bias in the same direction and neither can be quantified from within the registry, which contains only cases that were referred. The appropriate test is external triangulation: comparison of the registry against civil death-registration data for the identical catchment and period would estimate the number of suicides certified outside the medico-legal system and bound the size of the effect. That linkage has not, to our knowledge, been performed for these counties; it is identified as a priority in Section 6 and is a necessary condition for interpreting the reported proportions as population parameters rather than as characteristics of the ascertained series.

### 2.4. Variables and Outcome Definition

The binary outcome was the method of death, dichotomised as hanging (=1) versus all other methods (=0). Eleven predictors were used: age (continuous), sex, urban/rural residence, marital status, educational level (ordinal, 1–5), occupational status, history of psychiatric pathology, acute ethanol intoxication at autopsy, prior suicide attempts, presence of a suicide note, and county. All clinical predictors were taken directly from the medico-legal record.

### 2.5. Missing Data and Sensitivity Analysis

Missingness was substantial for two clinical fields, documented psychiatric history (68.1%) and quantified blood-alcohol concentration (76.5%), reflecting selective ante-mortem documentation and selective toxicological sampling rather than random loss. Missing values are therefore presumed Missing Not At Random (MNAR). For the primary analysis, continuous variables were imputed by the median and binary variables by the mode prior to model training; this introduces a conservative bias toward the null for the affected predictors, which is treated transparently as a limitation and addressed through the sensitivity analysis of Section Tipping-Point Sensitivity Analysis for Undocumented Clinical Fields. Multiple imputation by chained equations (MICE) was considered and is structurally infeasible for this registry rather than merely unperformed: the two affected fields record only positive findings—every non-missing entry is a positive diagnosis or a positive quantified concentration, and no explicit negative is ever recorded—so the absence of an entry is an inseparable mixture of “verified absent” and “not documented”. An imputation model trained exclusively on positive observed values would impute every missing entry as positive, and the procedure would collapse rather than inform. This is a structural property of single-source forensic registries, not an implementation choice.

#### Tipping-Point Sensitivity Analysis for Undocumented Clinical Fields

The appropriate robustness check for this missingness structure is a tipping-point (delta-adjustment) analysis under an explicit MNAR mechanism. A fraction q of the undocumented (blank, coded 0) entries of psychiatric history and blood-alcohol concentration was randomly reassigned to positive, for q = 5%, 10%, 20%, 30% and 50%, with repeated random reassignment at each level (60 replicates for the regression models, 25 for the random-forest importance ranking), and all models were re-estimated at every replicate. Three quantities were monitored: the identity of the two leading predictors in the OOB permutation-importance ranking, and the direction of the odds ratios for psychiatric history and for acute ethanol intoxication. Full results are reported in Supplementary Table S1 and summarised in Section 3.3.

### 2.6. Supervised Models, Choice of Algorithms and Hyperparameter Specification

Two classifiers were developed. The reference model was L2-regularised (ridge) logistic regression. The primary ML model was a random forest of 200 trees, with √p candidate predictors evaluated at each split and a minimum leaf size of three observations. Random forest was selected for its capacity to capture non-linear interactions and non-additive predictor relationships without the linearity and independence assumptions of logistic regression, and for its robust permutation-based importance estimation.

Hyperparameter specification. All hyperparameters were fixed a priori and were not tuned (200 trees, √p candidate predictors per split, minimum leaf size of three, default ridge penalty); no grid, random or Bayesian search was performed at any stage. With 23 minority-class events, any tuning conducted inside the reported cross-validation is optimistically biassed, and a nested search would leave roughly two minority events per inner validation fold, so the selection criterion could not distinguish signal from noise. Fixed conventional defaults yield a conservative, uncontaminated estimate; the full reasoning is given in Supplementary Methods S1.1.

Range of algorithms. The model set was restricted by design to one penalised linear reference and one non-linear ensemble; gradient-boosted ensembles and support-vector machines were considered and not pursued. The governing quantity is the minority-class count (*n* = 23), corresponding to roughly two events per variable against eleven predictors—an order of magnitude below the conventional minimum for stable estimation [22,23]—and flexible learners are markedly more data-hungry than penalised regression for dichotomous endpoints [24,25]. The close agreement of the two contrasting model families (ΔAUC = +0.018) is itself informative; algorithm expansion is conditional on enlarging the minority class through multicentre pooling (Section 6). The full derivation is given in Supplementary Methods S1.2.

### 2.7. Validation Strategy, Choice of k, and the Decision Not to Correct Class Imbalance

Both models were evaluated by 10-fold stratified cross-validation, preserving the outcome ratio in every fold. Performance was summarised by accuracy, AUC-ROC, sensitivity and specificity (mean ± standard deviation across folds). Because the outcome was severely imbalanced (94.2% hanging), accuracy is uninformative; a trivial classifier predicting “hanging” for every case would attain 94.2% accuracy, so AUC-ROC was pre-specified as the primary metric, consistent with established guidance for learning from imbalanced data. AUC values were interpreted using conventional thresholds (0.70–0.80 acceptable; 0.80–0.90 excellent; >0.90 outstanding).

Number of folds. Ten folds were retained rather than five because the binding constraint at 23 events is training-set information, not test-set precision: five folds would withhold twenty per cent of the data from every training set, and the resulting pessimistic bias outweighs the variance reduction in the test folds [26]. The consequence of the small per-fold minority count is a wide AUC estimate, not a biassed one, and it is reported as such (±0.181 and ±0.192). The full trade-off analysis is given in Supplementary Methods S1.3.

Class-imbalance correction. No imbalance correction (oversampling, SMOTE, class weighting or cost-sensitive learning) was applied, as a pre-specified decision. AUC, the pre-specified primary metric, is invariant to the class ratio, and imbalance corrections do not improve discrimination while systematically degrading calibration [27,28]; the apparent specificity gain is obtainable from the uncorrected model by shifting the classification threshold. More fundamentally, the 94.2% hanging proportion is the principal epidemiological finding of the paper, and the 23 non-hanging deaths are the real, complete count of such deaths in this catchment: synthesising additional minority cases would simulate away the very signal the study reports. The full reasoning, including the SMOTE-specific objections for binary features, is given in Supplementary Methods S1.4.

### 2.8. Predictor Importance, Effect-Size Uncertainty and Model Interpretation

Predictor importance was quantified by out-of-bag (OOB) permutation importance from a random forest trained on the full sample, complemented by mean L2 logistic-regression coefficients (with odds ratios) across folds and by Spearman rank correlations with the outcome. Positive OOB importance indicates a useful predictor; values at or below zero indicate that permuting the variable does not degrade or marginally improves performance, signalling noise or redundancy with stronger correlated predictors. Ninety-five percent confidence intervals for the odds ratios were obtained by stratified non-parametric bootstrap of the full estimation procedure: 2000 resamples drawn with replacement within each outcome class, preserving the 372/23 structure, with the interval taken from the 2.5th and 97.5th percentiles of the resampled coefficient distribution. The cross-validation folds themselves cannot supply these intervals, because folds share approximately 90% of their training data pairwise and the between-fold variability of a coefficient measures algorithmic stability rather than sampling uncertainty.

Model-agnostic post hoc explanation methods (SHAP, partial-dependence plots) were considered [29] and not applied: with nine of eleven predictors binary, their principal advantages do not engage, and attributions estimated against 23 minority-class events would communicate a precision the data do not support. Three mechanistically independent importance estimates—permutation-based, model-based and rank-correlational—are reported instead, and their mutual agreement is treated as the interpretability evidence; the full reasoning is given in Supplementary Methods S1.5. SHAP-based interpretation is stated in Section 6 as a next step once the minority class has been enlarged.

### 2.9. Unsupervised Clustering: Rationale, Procedure and Stability of the Partition

Rationale for an unsupervised stage on labelled data. The supervised and unsupervised stages answer different questions: the classifiers ask which recorded variables carry information about the method of death, while the clustering asks whether the 395 victims constitute a single population or several, without reference to any outcome. A supervised model conditioned on a 94.2–majority outcome is by construction dominated by the majority profile, so a small, internally coherent subgroup distinguished on variables other than the target is precisely what it cannot surface; withholding the label is the condition under which such a subgroup becomes visible. The two stages are complementary—the supervised branch establishes which variables matter, the unsupervised branch establishes for whom (Figure 1)—and the full argument is given in Supplementary Methods S1.6.

Latent structure was examined by K-means clustering on z-score–standardised features, evaluated for k = 2 to 7 with 50 random restarts and squared Euclidean distance. The optimal number of clusters was selected by maximising the mean silhouette coefficient, confirmed by the elbow method on within-cluster sum of squares (WCSS). Silhouette values were interpreted per established bands (0.50–0.70 reasonable structure; >0.70 strong structure). Between-cluster differences were tested with the chi-square test for categorical variables and the independent-samples *t*-test for age.

K-means on a predominantly binary/ordinal feature set is a recognised simplification: Gower-dissimilarity-based partitioning, k-modes and latent class analysis are the standard alternatives for mixed feature types [30,31,32]. They were not adopted here for the geometric reason set out below, but the choice is acknowledged as one among defensible alternatives rather than as uniquely correct.

Stability of the retained partition. Two considerations bear on the stability of the two-cluster solution. The first is the convergence of two independent selection criteria: the silhouette coefficient peaks sharply at k = 2 (0.707) and collapses at k = 3, while the elbow criterion on WCSS concurs. The second derives from the geometry of the partition itself: Cluster 2 is separated from Cluster 1 on two variables that take the value zero in every single member of Cluster 1—prior suicide attempts (57.9% vs. 0.0%) and suicide note (68.4% vs. 0.0%)—so the boundary coincides with a region of feature space that is empty by construction, and any partitioning method that respects the observed geometry must recover it.

As an empirical robustness check, Ward-linkage hierarchical clustering was run on the identical standardised feature matrix; it reproduced the K-means partition exactly (adjusted Rand index = 1.000; identical assignment of all 395 cases; cluster sizes 376/19). This concordance was expected given the geometry above, so it confirms the internal stability of the partition without establishing its generality. A Gaussian mixture model was not fitted, because its multivariate-normal component densities are undefined for a predominantly binary feature set. The status of the two-cluster partition as a generalisable typology can be established only by replication in an independent registry, stated as a required next step in Section 6, at which stage formal resampling-based stability assessment [33] becomes informative. The extended reasoning is given in Supplementary Methods S1.7.

### 2.10. Software

All analyses were performed in MATLAB R2025a (Statistics and Machine Learning Toolbox). Random number generation was seeded to a fixed value so that fold assignment, clustering restarts, bootstrap resampling and the sensitivity-analysis reassignments are exactly reproducible.

### 2.11. Ethics

The study used fully anonymised, de-identified medico-legal records from a population-based registry; no living participants were involved and no individual identifiers were processed. Forensic autopsy is legally mandated under the Romanian Code of Criminal Procedure, providing the lawful basis for case ascertainment. The study was conducted in accordance with the Declaration of Helsinki (2013 revision) and applicable data-protection law; individual informed consent was not applicable. Institutional ethics approval was obtained from the Ethics Committee of the Brăila County Emergency Clinical Hospital, Brăila, Romania (approval no. 18 dated 30 July 2026).

### 2.12. Reporting

The manuscript adheres to the TRIPOD+AI statement (2024) for prediction-model reporting and to the STROBE statement for observational studies; completed checklists are provided as Supplementary Material.

## 3. Results

### 3.1. Population Characteristics

Table 1 summarises the sociodemographic and clinical profile of the 395 victims. The study population was overwhelmingly male (87.1%), giving a male-to-female ratio of 6.7:1, markedly higher than the Western European average of 3–4:1; the ascertainment mechanisms that may contribute to this excess are set out in Section 2.3. The mean age was 54.1 years (SD 15.8; range 15–97) and 73.2% of victims were rural. Hanging accounted for 94.2% of deaths. Documented psychiatric history (31.9%) and acute ethanol intoxication (23.5%) were recorded well below the prevalences reported in clinical meta-analyses, consistent with the documented under-recording inherent to forensic data.

### 3.2. Supervised Model Performance

Table 2 presents cross-validated performance. Random forest achieved AUC = 0.865 ± 0.181 and logistic regression AUC = 0.847 ± 0.192; both fall within the “excellent” discrimination band. The modest RF advantage (ΔAUC = +0.018) is consistent across folds. Sensitivity was very high for both models (0.995–0.997): fewer than one hanging case in two hundred would be misclassified, a result of practical relevance for medico-legal screening. Specificity was limited (0.233–0.367) with large fold-to-fold variability—the direct mathematical consequence of the 23-case minority class, not a modelling error; the values correspond to the default 0.5 classification threshold and can be traded against sensitivity by moving that threshold, without refitting (Section 2.7). Accuracy (0.949–0.959) is reported for completeness but, as noted, is non-discriminating in this setting because a trivial majority-class classifier attains 94.2%.

In practical terms, the reported specificity implies that at the default 0.5 threshold approximately two-thirds to three-quarters of the non-hanging cases—roughly 15 to 18 of the 23—would be labelled as hanging by the classifiers. This is the operating characteristic of a screening orientation dominated by the majority class, not a property of the fitted probabilities: because the operating point is a reporting decision rather than a modelling one (Section 2.7), the threshold can be lowered to recover specificity at a quantified cost in sensitivity, without refitting either model. Any application that prioritises the detection of non-hanging deaths—for example, flagging cases for extended toxicological work-up—would operate the same models at a lower threshold.

### 3.3. Predictor Importance

Table 3 ranks the predictors in descending order of out-of-bag permutation importance. Prior suicide attempts dominated (OOB importance 0.959—almost twice the second-ranked variable), with a strong negative Spearman correlation (ρ = −0.549, *p* < 0.001) and an odds ratio of 0.496 (95% CI 0.25–0.72): their presence approximately halved the odds of hanging as the final method. The presence of a suicide note ranked second (importance 0.575; ρ = −0.439, *p* < 0.001; OR = 0.549, 95% CI 0.34–0.76). Acute ethanol intoxication (ρ = −0.168, *p* = 0.001) and psychiatric history (ρ = −0.131, *p* = 0.009) were the only additional variables reaching classical statistical significance; the negative OOB importance of psychiatric history reflects functional redundancy with the two leading predictors rather than absence of association. The bootstrap confidence intervals (Section 2.8) excluded unity for exactly the three predictors with significant rank correlations of the same sign—prior attempts, suicide note and acute ethanol intoxication—and crossed unity for all others, including psychiatric history (OR 0.774, 95% CI 0.43–1.32), which is quantitatively consistent with its redundancy with the two leading predictors. The width of the intervals is the direct expression of the 23-event minority class and should temper any reading of the point estimates as precise. Sex, residence, marital status, county and employment did not discriminate by method, an interpretable null indicating demographic homogeneity within this hanging-dominated population. The three importance estimates, permutation-based, model-based and rank-correlational, agree on the identity and ordering of the two leading predictors, which is the convergent-estimator evidence described in Section 2.8.

The tipping-point sensitivity analysis (Section Tipping-Point Sensitivity Analysis for Undocumented Clinical Fields; Supplementary Table S1) confirmed that these findings are stable under plausible degrees of clinical under-documentation: the identity of the two leading predictors was preserved in 100% of replicates at every reassignment level up to q = 50%, the direction of the ethanol odds ratio in 100% of replicates at every level, and the direction of the psychiatric-history odds ratio in at least 90% of replicates up to q = 30%, degrading only under the epidemiologically implausible q = 50% scenario, which would imply an overall psychiatric-history prevalence near two-thirds of the study population.

### 3.4. Unsupervised Clustering

K-means yielded an unambiguous two-cluster solution; given the size of the minority cluster, the result is reported as exploratory and hypothesis-generating. The silhouette coefficient peaked sharply at k = 2 (0.707, strong structure) and collapsed to 0.263 at k = 3 (Table 4); the elbow criterion concurred (WCSS dropping 10.9% from k = 2 to k = 3, then only marginally thereafter). The convergence of both criteria is a robustness guarantee that the two clusters are genuine, not algorithmic artefacts. The partition was identical across all 50 random restarts, and Ward-linkage hierarchical clustering on the same standardised features reproduced it exactly (adjusted Rand index = 1.000; identical assignment of all 395 cases; Section 2.9).

The two clusters (Table 5) were profoundly asymmetric. Cluster 1 (*n* = 376; 95.2%) reproduced the dominant regional profile—rural, male, middle-aged, no documented prior attempts or notes, hanging in 97.1%—and served as an implicit data-quality check against established epidemiology. Cluster 2 (*n* = 19; 4.8%) was the central, exploratory finding: a younger subgroup (42.4 vs. 54.7 years; Δ = −12.3) distinguished simultaneously on five dimensions—prior attempts (57.9% vs. 0%), suicide note (68.4% vs. 0%), psychiatric comorbidity (52.6% vs. 30.9%), method diversity (hanging only 36.8% vs. 97.1%) and reduced employment (15.8% vs. 33.0%). All differences were significant (*p* < 0.001). Two of the discriminating variables take the value zero in every member of Cluster 1, so the boundary between the clusters lies in an empty region of the feature space rather than at a distance threshold, the structural reason for the stability discussed in Section 2.9.

## 4. Discussion

To our knowledge, this is the first machine learning analysis of a Romanian forensic suicide registry and one of the very few applied to medico-legal suicide data anywhere [15]. Three findings stand out: an exceptionally high hanging proportion (94.2%), an internally consistent set of dominant predictors led by prior suicide attempts and suicide notes, and the unsupervised isolation of a small but clinically distinct high-risk subgroup.

The 94.2% hanging proportion is the strongest epidemiological signal in the dataset. It exceeds the pan-European average of roughly one half and even the elevated values (70–80%) reported for high-hanging Eastern European countries, and it surpasses earlier Romanian forensic descriptions [2,34,35]. The comparison must be read with the ascertainment caveat of Section 2.3 in mind: the proportion is computed over cases referred to the medico-legal system, and any suicide certified as a natural death without forensic referral would, by its nature, be more likely to involve a non-hanging method. The direction of that bias is upward, so the observed figure is best treated as an upper bound on the true regional proportion, while remaining high on any plausible adjustment. This dominance has direct methodological consequences: it generates severe class imbalance, which is why AUC, not accuracy, anchors the analysis, and why the limited specificity is an expected statistical artefact rather than a failure of modelling [21,36]. Paradoxically, the minority “other methods” class concentrates the cases of greatest preventive interest, as the clustering results make clear.

The supervised models confirmed that routinely recorded forensic variables carry genuine predictive information about the method of death (RF AUC 0.865; LR AUC 0.847), both in the “excellent” discrimination band. This performance is competitive with, and at the upper end of, the range reported in registry-scale machine learning studies and recent systematic reviews, where random-forest models consistently achieve AUC values between approximately 0.80 and 0.89 [8,9,10,11,12,15]. The modest RF advantage over logistic regression is less important than Random Forest’s robust, assumption-free permutation importance, which provided the interpretability backbone of the analysis [19]. The narrowness of that advantage is itself consistent with the broader methodological literature, in which flexible learners applied to tabular clinical data with modest event counts rarely outperform penalised regression by a margin exceeding the sampling variability [24]; it is one reason we did not expand the model set further (Section 2.6).

That prior suicide attempts emerged as the dominant predictor is fully consistent with the international evidence that previous self-harm is among the strongest risk factors for completed suicide [37,38]. The negative association with hanging indicates that attempters more often die by slower, alternative methods, plausibly those that allow time for communication and discovery, consistent with the parallel prominence of suicide notes [39]. The under-representation of notes in the full sample (3.5% vs. the international 15–40%) is itself a citable forensic observation [39], likely reflecting cultural, educational and documentation factors in a predominantly rural Eastern European population.

The clustering result is the study’s principal contribution to prevention. Cluster 2 would not have been visible to frequency tables or logistic regression; it required unsupervised learning [20,21]. Its defining feature, prior attempts in 57.9% versus 0% in the dominant profile, means that the majority of these victims had already contacted the medico-legal, psychiatric or emergency systems before death. In other words, the subgroup is prevention-relevant: its members were in documented contact with services before the fatal act, which defines an auditable target for the existing structured post-attempt follow-up recommended by the World Health Organization and current suicide-prevention guidelines [4], not a demonstrated missed intervention. The younger age (a loss of roughly twelve years of life relative to the dominant profile), higher psychiatric comorbidity and greater method diversity reinforce its interpretation as recurrent suicidal behaviour [38].

This interpretation is convergent with the typological literature on completed suicide, which the machine learning framing of the present study should not obscure. Latent class analysis of 28,703 suicide decedents in the US National Violent Death Reporting System identified classes defined by co-occurring mental-health, substance-related and prior-attempt profiles and concluded that most decedents fall into recognisable risk-factor patterns requiring better-connected services [30]. A cluster analysis of suicide deaths in Toronto likewise separated subgroups distinguished by psychiatric illness and prior suicidal behaviour from a larger residual profile [40], and contemporary models of suicidal behaviour treat the transition from ideation and attempt to death as phenotypically structured rather than homogeneous [41]. Cluster 2 reproduces, in an exhaustive autopsy-confirmed registry and at regional scale, the recurrent-attempt/psychiatric-comorbidity phenotype these studies describe; the convergence of typologies obtained by different unsupervised methods, in different jurisdictions and data systems, argues that the subgroup is a real clinical entity rather than a registry artefact, while its precise boundaries in this dataset remain exploratory pending external replication.

A qualification is required at this point, because the inference that follows from these data is narrower than it may appear and the distinction matters for what can legitimately be recommended. Nothing in this study establishes that post-attempt suicide prevention is absent in Romania. Structured follow-up after a suicide attempt is a standard component of suicide-prevention practice across European health systems, precisely because the elevated risk of repetition has been recognised for decades, and a forensic registry is in principle incapable of demonstrating the absence of such provision: it records deaths, not the services the decedents did or did not receive, and it contains no variable indicating whether follow-up was offered, accepted or completed. What these data do establish is different and, we would argue, more useful. Of the nineteen victims constituting Cluster 2, a majority had a documented prior attempt, which means that a majority had already been in contact with the emergency, psychiatric or medico-legal system before the fatal act, and that this contact did not avert the outcome. That is a statement about reach and continuity of care in a particular catchment, not about the existence of a policy. The appropriate inference is therefore not that a post-attempt prevention policy should be created, but that the local implementation of the existing one deserves audit: the linkage between emergency presentation for self-harm and subsequent specialist follow-up in the Galați–Brăila region is measurable through routine health-service data, has not to our knowledge been measured, and constitutes the natural successor study. We have reframed the recommendation in the Conclusions accordingly, and we regard the identification of a concrete, countable target group, rather than a general call for policy, as the specific contribution of the forensic-ML approach.

Strengths include the population-based, exhaustive design with autopsy-confirmed cause and manner of death, the seven-year window, and the dual supervised–unsupervised framework reported under contemporary guidelines (TRIPOD+AI, STROBE) [42,43]. A further strength is methodological transparency about what was deliberately not done [44]: the fixed hyperparameters, the restricted model set, the retention of ten folds and the decision not to correct the class imbalance are each pre-specified and argued in Section 2 and Supplementary Methods S1, so that the reported performance is a conservative estimate rather than a selected one; the bootstrap effect-size intervals, the tipping-point sensitivity analysis and the Ward-linkage concordance check added in revision quantify, rather than assert, the robustness of the findings.

## 5. Limitations and Future Research Directions

**Figure 3:**
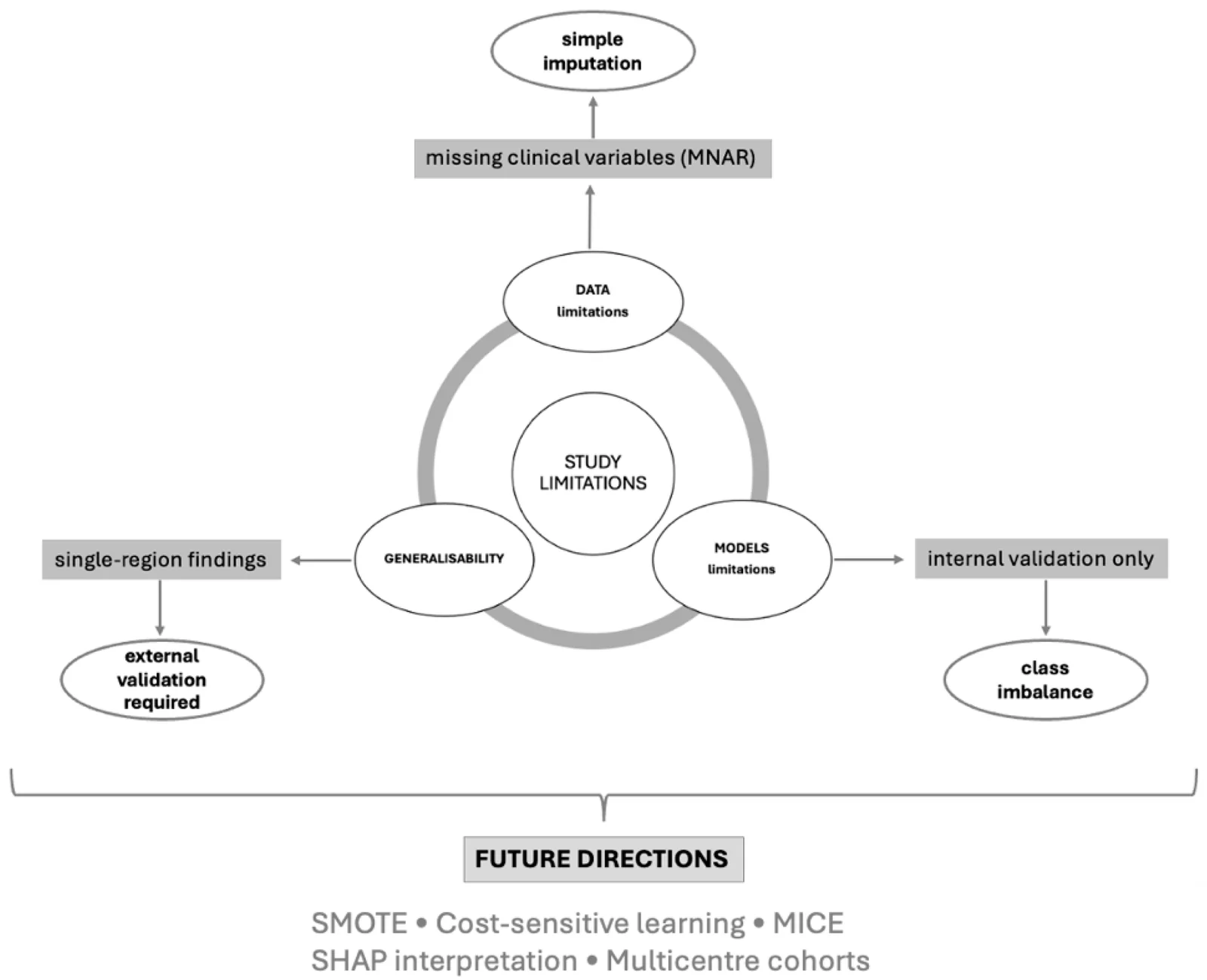
Conceptual overview of the study limitations and future research directions.

This study has several limitations (Figure 3) that should be acknowledged, as follows:

First, the severe class imbalance widens the confidence intervals for AUC and destabilises specificity; the pre-specified reasons for not correcting it are given in Section 2.7 and Supplementary Methods S1.4. This remains a limitation notwithstanding the justification: the reported specificity is imprecise and the minority class is too small to support subgroup analysis within it. Oversampling, cost-sensitive learning and a redefinition of the target toward more balanced outcomes should be tested once the minority class has been enlarged through multicentre pooling.

Second, the range of algorithms is restricted to one penalised linear model and one bagged ensemble, for the events-per-variable reasons given in Section 2.6 and Supplementary Methods S1.2; the consequence is that we cannot exclude the possibility that a more flexible learner would extract additional signal, only that the present sample cannot demonstrate it reliably. This comparison is a defined next step conditional on a larger pooled minority class.

Third, key clinical variables are under-recorded and presumed MNAR, and because the registry records only positive findings, multiple imputation is structurally infeasible (Section 2.5). The tipping-point sensitivity analysis (Section Tipping-Point Sensitivity Analysis for Undocumented Clinical Fields; Supplementary Table S1) shows that the principal findings are stable across all plausible degrees of under-documentation; the residual limitation is that the recorded prevalences of psychiatric history and alcohol involvement remain lower bounds on the true values, and the mode-imputed primary estimates remain conservatively biassed toward the null.

Fourth, ascertainment into the forensic system depends on a death being flagged as suspicious before the prosecutor is involved (Section 2.3). Suicides certified as natural deaths without forensic referral are invisible to this design, and because such deaths are more likely to be female and non-violent in method, the male-to-female ratio and the hanging proportion reported here should be treated as upper bounds. Linkage against civil death-registration data for the same catchment would bound the magnitude of this effect and is a priority.

Fifth, a marital-status field became constant after imputation and was excluded; it requires careful recoding.

Sixth, the models were internally validated only; external validation in adjacent counties is the necessary next step, alongside model-agnostic interpretation (e.g., SHAP) and multicentre expansion to enlarge the minority class. Repeated stratified cross-validation and bootstrap optimism correction would narrow the reported internal intervals but cannot substitute for an independent dataset.

Seventh, the two-cluster partition is stable within this dataset, but its status as a generalisable typology is not established. Internal criteria and comparisons between clustering algorithms cannot establish it (Section 2.9); the exact Ward-linkage concordance reported in Section 3.4 confirms internal stability, not generality; only replication of the partition in an independent registry can, and formal resampling-based stability assessment [33] becomes informative at that stage.

Finally, the findings describe a specific south-eastern Romanian region and should be generalised only as hypotheses.

## 6. Conclusions

Applied to a complete forensic suicide registry, machine learning reproduced the established epidemiology of the region and, going beyond classical statistics, isolated an exploratory but clinically coherent high-risk subgroup characterised by prior attempts, suicide notes and psychiatric comorbidity, stable under both an independent partitioning method and a formal sensitivity analysis for the undocumented clinical fields. The analysis demonstrates that routinely recorded medico-legal variables carry meaningful predictive structure and supports integrating ML into Romanian forensic research and into the regional targeting of post-attempt suicide prevention. We do not conclude that such prevention is absent: structured post-attempt follow-up is standard European practice, and a death registry cannot evidence its absence. We conclude, more narrowly and more testably, that a majority of the victims in the high-risk cluster had already entered the emergency, psychiatric or medico-legal system before the fatal act, and that the continuity of care between that first contact and specialist follow-up in the Galați–Brăila catchment is measurable, has not been measured, and should be audited. The contribution of the forensic machine learning approach is that it converts a general preventive principle into a specific, enumerable target group within a defined jurisdiction.

## Figures and Tables

**Table 1:** Characteristics of the study population (N = 395), Galați–Brăila, 2018–2024. * Clinical psychological-autopsy meta-analyses report 87–91% lifetime psychiatric disorder; the lower registry figure reflects incomplete ante-mortem documentation, not true prevalence. Missing clinical values were imputed (median/mode) prior to ML analysis. The reported proportions characterise the ascertained forensic series; see Section 2.3 for the ascertainment mechanisms that bound their interpretation as population parameters.

Variable	N (%)/Value	International Reference
Total cases	395 (100%)	—
Male sex	344 (87.1%)	WHO Europe ~75–80%
Female sex	51 (12.9%)	M:F = 6.7:1 vs. EU 3–4:1
Age, mean (SD; range)	54.1 (15.8; 15–97)	EU 45–55 years
Rural residence	289 (73.2%)	Romania ~57–65%
Urban residence	106 (26.8%)	—
Galați county	267 (67.6%)	—
Brăila county	128 (32.4%)	—
—Method—		
Hanging (ML target)	372 (94.2%)	EU ~50%; E. Europe ~70–80%
Other methods	23 (5.8%)	Minority class
—Clinical—		
Psychiatric history	126 (31.9%)	Meta-analyses 87–91% *
Acute ethanol intoxication	93 (23.5%)	EU 20–30%
Prior suicide attempts	28 (7.1%)	Literature 10–20%
Suicide note present	14 (3.5%)	International 15–40%
—Missing data—		
Psychiatric history (missing)	269 (68.1%)	Structural under-recording
Blood-alcohol (missing)	302 (76.5%)	Selective autopsy sampling

**Table 2:** Cross-validated performance of the supervised models (10-fold stratified CV, N = 395; mean ± SD). AUC-ROC was the pre-specified primary metric under class imbalance. The low specificity is an expected consequence of the minority class (*n* = 23) and of the default 0.5 classification threshold; no class-imbalance correction was applied (Section 2.7).

Model	Accuracy	AUC-ROC	Sensitivity	Specificity
Logistic regression (L2)	0.959 ± 0.024	0.847 ± 0.192	0.997 ± 0.009	0.367 ± 0.350
Random forest (200 trees)	0.949 ± 0.026	0.865 ± 0.181	0.995 ± 0.011	0.233 ± 0.344
Difference (RF − LR)	−0.010	+0.018	−0.002	−0.134
Interpretation	—	Excellent (>0.80)	Excellent	Imbalance-limited

**Table 3:** Predictor importance for the method of death. RF OOB permutation importance: values > 0 indicate a useful predictor; values ≤ 0 indicate noise or redundancy. Logistic-regression β and OR are means across 10 CV folds (L2 penalty); OR < 1 = associated with non-hanging methods; 95% CIs for OR from stratified non-parametric bootstrap of the same L2 estimation procedure (2000 resamples, percentile method; Section 2.8). A constant marital-status field after imputation (importance 0.000, ρ undefined) was excluded from final models. Rows have been reordered so that the rank column is strictly monotone in OOB importance; the previous ordering contained a transposition (ranks 4–7) and no values have been changed.

Rank Variable	RF OOB Imp.	β (LR)	OR	95% CI (OR)	Spearman ρ	*p*
1 Prior suicide attempts	0.959	−0.702	0.496	0.25–0.72	−0.549	<0.001
2 Suicide note present	0.575	−0.599	0.549	0.34–0.76	−0.439	<0.001
3 Educational level	0.187	−0.496	0.609	0.31–1.07	−0.085	0.091
4 Alcohol present	0.098	−0.722	0.486	0.25–0.82	−0.168	0.001
5 Age	0.091	+0.360	1.433	0.89–2.73	0.097	0.055
6 Male sex	0.082	−0.256	0.775	0.37–1.25	0.001	0.984
7 Galați county	0.056	+0.323	1.381	0.77–2.62	0.036	0.479
8 Rural residence	−0.109	+0.280	1.323	0.76–2.30	0.045	0.377
9 Psychiatric history	−0.131	−0.256	0.774	0.43–1.32	−0.131	0.009
10 Employed	−0.158	−0.019	0.981	0.53–1.95	0.008	0.877

**Table 4:** Selection of the optimal number of clusters by silhouette score and the elbow method (WCSS). A silhouette of 0.707 at k = 2 indicates strong, well-separated structure. Silhouette coefficients are computed on squared Euclidean distances, consistent with the clustering criterion; on unsquared Euclidean distances, the k = 2 value is 0.48, with the same ordering across k.

k	Silhouette	WCSS	Interpretation
2	0.707	3432.7	Optimal—strong structure
3	0.263	3057.3	—
4	0.287	2672.0	—
5	0.285	2439.4	—
6	0.283	2217.8	—
7	0.258	2036.5	—

**Table 5:** Profiles of the two clusters (K-means, k = 2, 50 restarts, silhouette = 0.707). Differences significant at *p* < 0.001 (chi-square for categorical variables; *t*-test for age). ↑/↓ denote distinctively higher/lower values relative to Cluster 1.

Variable	Cluster 1—Standard (N = 376; 95.2%)	Cluster 2—High-Risk (N = 19; 4.8%)
Mean age (years)	54.7 (SD 15.8)	42.4 (SD 20.8)—Δ −12.3
Male sex	87.2%	84.2%
Rural residence	72.9%	78.9%
Employed	33.0%	15.8%
Psychiatric history	30.9%	52.6% ↑
Alcohol present	23.1%	31.6% ↑
Prior suicide attempts	0.0%	57.9% ↑↑↑
Suicide note present	0.0%	68.4% ↑↑↑
Hanging (method)	97.1%	36.8% ↓↓↓
Proposed label	Typical regional victim	Recurrent suicidal behaviour

## Data Availability

The anonymised dataset analysed is available from the corresponding author on reasonable request, subject to forensic data-governance approval.

## Supplementary Materials

The following supporting information can be downloaded at: https://www.mdpi.com/article/10.3390/diagnostics16162615/s1, Table S1: Tipping-point sensitivity analysis: proportion of replicates in which each finding is preserved, by reassignment fraction q.
