# Supplementary material for: Machine Learning Identifies High-Risk Suicide Profiles in a Population-Based Forensic Registry
Source: Diagnostics (Basel). 2026 Aug 18;16(16):2615. doi: 10.3390/diagnostics16162615 (PMC13511942; doi:10.3390/diagnostics16162615)
Supplement: Supplementary file 1 [file diagnostics-16-02615-s001.zip › diagnostics-4464381-supplementary.pdf]

Supplementary Methods S1

Manuscript: Machine Learning Identifies High-Risk Suicide Profiles in a Population-Based Forensic Registry (diagnostics-4464381).

This file contains the full methodological derivations condensed out of Sections 2.5–2.9 of the main text in revision, together with the design and results of the tipping-point sensitivity analysis (Supplementary Table S1).

S1.1. Hyperparameter specification

All hyperparameters were fixed a priori and were not tuned: 200 trees, √p candidate predictors per split, minimum leaf size of three, and the default ridge penalty. No grid search, random search or Bayesian optimisation was performed at any stage. This was a deliberate design decision rather than an omission, and the reason is arithmetical. With 23 minority-class events, any hyperparameter search conducted inside the same cross-validation loop from which performance is subsequently reported yields optimistically biased estimates, because the folds have then been used twice, once to select and once to evaluate. The correct remedy is a nested search, but a nested loop would leave roughly two minority events in each inner validation fold, so the selection criterion would be estimated on a sample too small to distinguish signal from noise and the procedure would optimise the model against fold-assignment artefacts. Fixed conventional defaults produce an estimate that is conservative, in all likelihood slightly below the achievable optimum, but that is not contaminated by selection on the evaluation data. We consider a conservative honest estimate preferable to an optimistic tuned one at this sample size, and we state the values explicitly so that the analysis is exactly reproducible.

S1.2. Restriction of the model set

The model set was restricted, by design, to one penalised linear reference and one non-linear ensemble. Gradient-boosted ensembles (XGBoost, LightGBM, CatBoost) and support-vector machines were considered and not pursued. The quantity that governs this decision is not the total sample size (n = 395) but the number of minority-class events (n = 23), since it is the minority class that carries all the information available for discriminating the two outcomes. Against eleven candidate predictors this corresponds to approximately two events per variable, roughly an order of magnitude below the conventional minimum for stable estimation and far below the requirement derived from modern sample-size criteria for prediction models [31,32]. Boosted ensembles carry substantially more effective capacity than a random forest and, being sequential rather than bagged, lack the variance-averaging that makes random forests comparatively robust in small samples; simulation evidence shows that flexible learners are markedly more data-hungry than logistic regression for dichotomous endpoints [34], and a systematic review of clinical prediction modelling found no consistent performance advantage of machine learning over logistic

regression on tabular clinical data [33]. Adding four further algorithms to a 23-event outcome would multiply the number of reported comparisons without adding information: the between-algorithm differences so obtained would be smaller than, and statistically indistinguishable from, the fold-to-fold variability already visible in the reported standard deviations (± 0.181 and ± 0.192 AUC). Reporting them would give the appearance of a broader methodological assessment while in fact reducing the interpretability of the result. We therefore report two contrasting model families, one linear and penalised, one non-linear and ensemble-based, whose close agreement (ΔAUC = +0.018) is itself informative, and we treat algorithm expansion as conditional on enlarging the minority class through the multicentre pooling identified in Section 6 of the main text.

S1.3. Number of folds

The choice of ten rather than five folds requires justification, since stratification of 23 minority cases across ten folds places only two to three of them in each test fold. Reducing the number of folds does not solve this problem; it relocates it. Five-fold cross-validation would place approximately five minority cases in each test fold, a modest gain in per-fold precision, but would simultaneously withhold twenty per cent of the data from every training set, which at 23 events means fitting each model on roughly eighteen. The binding constraint in this dataset is training-set information, not test-set precision, and under that condition the pessimistic bias introduced by smaller training sets outweighs the variance reduction obtained in the test folds. Ten-fold cross-validation is the standard recommendation under precisely this bias–variance trade-off [37], and it is for this reason that we retain it. The consequence of the small per-fold minority count is not a biased AUC estimate but a wide one, and that consequence is reported rather than concealed: the fold-to-fold standard deviations of ± 0.181 and ± 0.192 are large by construction and are the honest expression of the uncertainty attaching to 23 events. Repeated stratified cross-validation and bootstrap optimism correction are the appropriate internal refinements and would narrow the reported interval without moving the point estimate; external validation on an independent registry is the only procedure that can establish generalisability, and neither internal method substitutes for it. Both are stated as required next steps in Section 6 of the main text.

S1.4. Class-imbalance correction

No imbalance correction, random oversampling, SMOTE, class weighting or cost-sensitive learning, was applied, and this was a pre-specified decision rather than an oversight. Three considerations govern it. First, the pre-specified primary metric is AUC-ROC, which is invariant to the class ratio and to any monotone rescaling of the predicted probabilities; simulation studies show that imbalance corrections do not improve discrimination while systematically degrading calibration, strongly overestimating the probability of minority-class membership [35,36]. Second, the gain in specificity that such corrections appear to deliver is obtainable from the uncorrected model simply by shifting the classification threshold, and without distorting the fitted probabilities [35]; the operating point is a reporting decision, not a modelling one, and the low specificity reported corresponds to the default 0.5 threshold rather than to a limitation of the fitted models. Third, and specific to this dataset, SMOTE generates synthetic minority observations by

interpolating between neighbouring cases in feature space. Nine of the eleven predictors here are binary or ordinal, so interpolation yields fractional values with no forensic referent, a synthetic record that is 0.4 male and carries 0.6 of a suicide note is not a case that could exist, and with only 23 minority cases the nearest-neighbour set of any seed observation spans a substantial fraction of the entire minority class, so the synthetic points are near-duplicates of their parents rather than new information. There is a further and more fundamental objection. The class imbalance in this study is not a data-collection artefact awaiting correction: the 94.2% hanging proportion is the principal epidemiological finding of the paper, and the 23 non-hanging deaths are the real, complete and irreducible count of such deaths in this catchment over seven years. Synthesising additional minority cases would consist of simulating away the very signal the study reports. Oversampling and cost-sensitive learning are retained as explicit future-work items, conditional on a pooled multicentre minority class of adequate size, at which point the trade-off changes and the comparison becomes informative.

S1.5. Post-hoc explanation methods (SHAP, partial dependence)

Model-agnostic post-hoc explanation methods, SHAP values and partial-dependence or accumulated-local-effect plots, were considered [38] and were not applied, for two reasons that are specific to this dataset rather than general reservations about the methods. The first is that their principal advantages do not engage. SHAP decomposes individual predictions and partial-dependence plots recover non-monotone marginal effect shapes; but with nine of eleven predictors binary, a partial-dependence curve degenerates to the two-point contrast that the odds ratios in Table 3 already report, and a SHAP summary reduces to a re-scaling of a ranking that is presented in full. The second is a matter of statistical honesty. SHAP attributions inherit the variance of the model they explain: estimated on a forest trained against 23 minority-class events, the attributions relevant to that class would be unstable across resamples, and displaying them with the visual authority of a beeswarm plot would communicate a precision the underlying data do not support. Three importance estimates obtained by three mechanistically distinct routes are therefore reported, permutation-based (OOB importance), model-based (penalised regression coefficients and odds ratios) and non-parametric (Spearman rank correlation), and their mutual agreement, visible in Table 3, is treated as the interpretability evidence. Convergent estimates from independent estimators are a stronger form of interpretability at this sample size than a single high-resolution attribution method. SHAP-based interpretation is stated in Section 6 of the main text as a next step once the minority class has been enlarged.

S1.6. Rationale for an unsupervised stage on labelled data

Since the dataset carries a label and the primary aim is prediction, the addition of an unsupervised analysis requires justification; the two are frequently and wrongly treated as alternatives, when in fact they answer different questions. The supervised models take the method of death as the target and ask which recorded variables carry information about it: the outcome is fixed in advance, and every result is conditional on that choice. The clustering takes no target at all and asks whether the 395 victims constitute a single population or several, a question about the structure of the victim

population, not about the method of death. The distinction is substantive rather than formal. A supervised model conditioned on an outcome that is 94.2% majority is by construction dominated by the majority profile; a small, internally coherent subgroup distinguished on variables other than the target is precisely what such a model cannot surface, because its members are absorbed into the majority class the model is optimising against. Cluster 2 is exactly that case: it is defined by the joint configuration of prior attempts, suicide note, age and psychiatric comorbidity, and it is identifiable without reference to the method of death at all. Withholding the label is therefore not a loss of information but the condition under which the subgroup becomes visible. The two stages are complementary in the strict sense, the supervised branch establishes which variables matter, the unsupervised branch establishes for whom, and their convergence on the same two dominant variables, reached by independent routes, constitutes mutual corroboration rather than duplication (Figure 1 of the main text). The clinical value added is the identification of an intervention target: a ranked predictor list does not by itself define a group of people who could have been reached, whereas a cluster does.

S1.7. Stability of the partition and alternative clustering methods

Two considerations bear on the stability of the two-cluster solution. The first is the convergence of two independent selection criteria. The silhouette coefficient peaks sharply at $k = 2$ (0.707, computed on squared-Euclidean distances, consistent with the clustering criterion) and collapses to 0.263 at $k = 3$, while the elbow criterion on WCSS concurs; a separation-based and a compactness-based criterion are not guaranteed to agree, and their agreement here argues against an algorithmic artefact. The second consideration is stronger and derives from the geometry of the partition itself. Cluster 2 is separated from Cluster 1 on two variables that take the value zero in every single member of Cluster 1 — prior suicide attempts (57.9% vs 0.0%) and suicide note (68.4% vs 0.0%). The boundary between the clusters therefore does not depend on a distance threshold, on centroid initialisation, or on any distributional assumption: it coincides with a region of feature space that is empty by construction, and any partitioning method that respects the observed geometry must recover it.

This geometry generated the a priori prediction that Ward-linkage hierarchical clustering on the same standardised features would reproduce the partition. In revision, the check was run rather than argued: Ward-linkage agglomerative clustering ($k = 2$) on the identical z-scored feature matrix reproduced the K-means partition exactly, with an adjusted Rand index of 1.000, identical assignment of all 395 cases, and identical cluster sizes (376/19). Because the concordance was expected on geometric grounds, it is reported as a confirmation of internal stability rather than as evidence of generality. A Gaussian mixture model was not fitted, since nine of eleven variables are binary and the multivariate-normal component densities the method assumes are not defined for them; a high concordance obtained under violated assumptions would carry the appearance of validation without its substance. Gower-dissimilarity-based partitioning, k-modes and latent class analysis are acknowledged as the standard alternatives for mixed binary/ordinal feature sets [40,43,44]. The status of the two-cluster partition as a generalisable typology is established by neither internal criterion nor method comparison, but only by replication in an independent

registry; formal resampling-based stability assessment [39] is appropriate at that stage, when a partition estimated on one dataset can be tested against another.

S1.8. Bootstrap confidence intervals for the odds ratios

Ninety-five percent confidence intervals for the odds ratios in Table 3 were obtained by stratified non-parametric bootstrap of the full estimation procedure. In each of 2,000 replicates, cases were resampled with replacement separately within the hanging (n = 372) and non-hanging (n = 23) classes, preserving the outcome structure; features were re-standardised and the L2-penalised logistic regression was refitted on each resample with the same penalty as the primary analysis. The interval for each coefficient was taken from the 2.5th and 97.5th percentiles of the resampled coefficient distribution and exponentiated. The cross-validation folds cannot supply these intervals: folds share approximately 90% of their training data pairwise, so the between-fold standard deviation of a coefficient measures algorithmic stability, not sampling uncertainty, and intervals constructed from it would be spuriously narrow — for the leading predictor, roughly one-fifth of the correctly derived width.

S1.9. Tipping-point sensitivity analysis for undocumented clinical fields

Multiple imputation by chained equations is structurally infeasible for this registry: the two high-missingness fields (documented psychiatric history, 68.1%; quantified blood-alcohol concentration, 76.5%) record only positive findings, with no explicit negative ever entered, so the absence of an entry is an inseparable mixture of "verified absent" and "not documented", and an imputation model trained exclusively on positive observed values would impute every missing entry as positive. The appropriate robustness check under this structure is a tipping-point (delta-adjustment) analysis with an explicit MNAR mechanism. A fraction q of the undocumented (blank, coded 0) entries of both variables was randomly reassigned to positive, for q = 5%, 10%, 20%, 30% and 50%; reassignment was repeated 60 times per level for the regression models and 25 times per level for the random-forest importance ranking, and all models were re-estimated at every replicate. Three quantities were monitored: (i) preservation of {prior suicide attempts, suicide note} as the top two predictors of the OOB permutation-importance ranking; (ii) the sign of the logistic-regression coefficient for acute ethanol intoxication; (iii) the sign of the coefficient for psychiatric history. Results are given in Supplementary Table S1. At q = 50%, the implied overall prevalence of psychiatric history approaches two-thirds of the study population, which is epidemiologically implausible for a documented-history variable; within the plausible range (q ≤ 30%) all three monitored quantities are stable.

Supplementary Table S1. Tipping-point sensitivity analysis: proportion of replicates in which each finding is preserved, by reassignment fraction q.

q (undocumented entries reassigned positive)	OOB top-2 {prior attempts, suicide note} preserved	Direction of ethanol OR preserved ($\beta < 0$)	Direction of psychiatric-history OR preserved ($\beta < 0$)
5%	100%	100%	100%
10%	100%	100%	100%
20%	100%	100%	98%

30%	100%	100%	90%
50%	100%	100%	80%
